# Towards Continuous Swim Leg Analytics in Olympic Triathlon: A Systematic Review of Sensor-Based Assessment Approaches in Open-Water Sports Contexts

**DOI:** 10.3390/s26072151

**Published:** 2026-03-31

**Authors:** Jannik Seelhöfer, Jürgen Wick, Maren Witt

**Affiliations:** 1 Department of Sports Biomechanics, Faculty of Sports Science, Leipzig University, 04109 Leipzig, Germany; biomechanik@uni-leipzig.de; 2 Department of Endurance Sports, Institute for Applied Training Science, 04109 Leipzig, Germany; iat@sport-iat.de

**Keywords:** triathlon, swimming, GNSS, sensors, race analysis

## Abstract

Global Navigation Satellite Systems (GNSS) offer precise movement analyses based on distance and speed in open-water sports. Despite the influence of swimming in triathlon, its performance analysis remains underdeveloped due to methodological limitations in capturing continuous data in aquatic environments. This review aimed to: (1) systematically analyse and compare the sensor-based technologies applied to open-water movement analysis, and (2) propose a framework for continuous GNSS-based assessment of triathlon swim performance. A systematic search was conducted prior to the 14 August 2025 across four databases (Web of Science, SPORTDiscus, PubMed, and SPONET). Studies were eligible if they analysed open-water sports using GNSS-based technologies for continuous movement or performance analysis. Studies limited to indoor swimming, inertial sensors, or non-sporting applications were excluded. Methodological quality and potential sources of bias were evaluated using a custom scheme based on GNSS reporting guidelines, as methodological heterogeneity precluded the application of standardised tools. Following screening and eligibility assessment, articles were analysed qualitatively. In total, 20 articles were included and focused on surfing, sailing, water skiing, windsurfing, kitesurfing, stand-up paddling (SUP), and swimming. Most studies focused on board- and sail-based sports, employed sampling frequencies between 1 and 15 Hz, and demonstrated substantial variability in device specifications and reporting quality. Different sensors and GNSS-derived variables were central to discipline-specific performance analysis. The strength of evidence is limited by the heterogeneous methodologies, and variable reporting quality. The proposed framework provides methodological guidance for implementing high-resolution GNSS-based monitoring in triathlon swimming to improve pacing analysis and race strategy development.

## 1. Introduction

In triathlon, race outcomes are strongly shaped by the opening swim leg, as early group formation and positional dynamics substantially influence subsequent cycling and running performance. However, precise data for systematically analysing swim pacing strategies and deriving practice-oriented training recommendations remain limited. Whereas cycling and running metrics are well established, swim pacing in triathlon remains poorly understood [1]. Traditionally, swim speed in triathlon has been derived from split times in combination with pre-measured course distances [2,3]. For example, Vleck et al. [3] used a video-timing system with calibrated segment distances determined via a handheld Global Navigation Satellite System (GNSS) device (Garmin eTrex Vista) and calibrated measuring ropes (buoy 1: 222 m; buoy 2: 496 m; swim exit: 693 m; two laps) to derive split-based pacing. These analyses demonstrated notable speed differences across the fixed sections. However, these approaches capture only a handful of fixed splits, limiting temporal resolution and tactical insight. Recently, open-water swimming research has begun to fill this void with higher-resolution splits and advanced tactical analytics [4,5]. Emerging GNSS-based continuous monitoring approaches in open-water swimming have begun to demonstrate the technical feasibility of such analyses [6]. Nevertheless, although recent studies provide important evidence regarding the feasibility of continuous GNSS-based monitoring in open-water swimming, a systematic integration of these insights into a triathlon-specific methodological framework has not yet been undertaken. Against this background, racecourse analysis in triathlon becomes essential for understanding the complex interplay of competition dynamics, athlete performance, and strategic decision-making. Such analyses make it possible to identify performance-relevant factors and thus provide a basis for targeted interventions against common challenges including inefficient pacing strategies, suboptimal course navigation, or tactical errors.

Sensor-based technologies such as GNSS and Inertial Measurement Units (IMU) enable detailed analyses of individual race dynamics by providing precise data on parameters like distance covered, velocity and acceleration. GNSS derives distance and speed through Doppler-shift effects and positional differentials [7], whereas IMU estimate velocity by integrating acceleration signals [8]. However, IMU-based estimates are prone to cumulative drift errors that can lead to substantial deviations over time [9,10]. For instance, Dadashi et al. [9] observed root mean squared errors of 0.11 m·s^−1^ and peak errors up to 0.18 m·s^−1^ in instantaneous velocity estimation in water, while Stamm et al. [10] reported an integration drift of 0.08 m·s^−1^ over a 40 s lap (0.002 m·s^−1^ per integrated second) with the sensor sitting at rest. Because distance remains a comparatively stable and error-resilient metric, it serves as the foundational parameter for race-course analysis.

Numerous open-water sports including sailing [11], surfing [12,13], and windsurfing [14] routinely deploy GNSS to analyse movement patterns in both training and competition, achieving high-resolution trajectory mapping and in-depth performance diagnostics. In triathlon, however, only one study to date has implemented a GNSS module for swimming [15], and, similar to earlier work by Vleck et al. [2,3], this research focused on a limited set of split-segment analyses rather than continuous course tracking. No systematic review has synthesised the methodological characteristics of GNSS applications across open-water disciplines with specific reference to the swim leg in triathlon. In particular, the role of sampling frequency, signal quality, sensor placement, and derived analytical metrics for continuous swim monitoring remain unclear. A structured methodological synthesis is therefore required to translate existing open-water GNSS applications into a triathlon-specific analytical framework.

Therefore, the objective of this systematic review is two-fold: (1) to systematically analyse and compare the breadth of sensor-based technologies applied to open-water movements and (2) to propose a customised analytical framework for continuous swim assessment in a triathlon, encompassing device selection criteria as well as measurement accuracy and precision.

## 2. Materials and Methods

The search and reporting processes in this systematic review were conducted and reported in accordance with the PRISMA 2020 guidelines to enhance the transparency and reproducibility of the study selection [16]. The review protocol was prospectively registered with the International Prospective Register of Systematic Reviews (PROSPERO; registration number CRD420251173256), and eligibility criteria, search strategy, and screening procedures were predefined prior to study selection.

### 2.1. Information Sources and Search Strategy

A systematic literature search was performed prior to the 14 August 2025 across four databases (Web of Science, SPORTDiscus, PubMed, and SPONET) to identify all methodological and technical approaches for the analysis of positional and movement data in open-water sports. The selected databases were chosen to ensure comprehensive coverage of sport science, biomechanics, and applied performance research. Search strings combined keywords for analytical intent (analysis OR evaluation OR data OR event OR movement OR detection OR performance OR assessment), positioning technologies (GPS OR GNSS OR sensor* OR microsensor OR wearable OR “global positioning system” OR “global navigation satellite system” OR position*), and relevant disciplines (swim* OR sail* OR windsurf* OR kitesurf* OR surfing OR surfer* OR triathlon OR “open water”), while explicitly excluding terms related to animal studies, biology, and paralympic sport (animal* OR fish OR biology OR paralympic). Only articles published in peer-reviewed, English-language journals within the Sport Sciences and Engineering research areas of Web of Science were considered. To ensure conceptual alignment with the tactical and navigational characteristics of triathlon swim legs, inclusion was deliberately limited to studies examining performance in open-water sports with non-linear course characteristics. After completion of the predefined systematic search period, one additional highly relevant study in open-water swimming was published. As this study appeared outside the predefined search window, it was not included in the formal systematic synthesis but is considered in the Discussion due to its direct relevance to the aims of the present systematic review.

### 2.2. Eligibility Criteria

The inclusion and exclusion criteria were developed based on the PICO(S) framework (Population, Intervention, Comparison, Outcome, and Study design) [17], with the study design (“S”) component included to support the targeted selection of relevant study types, see Table 1.

### 2.3. Selection Process and Data Extraction

**Figure 1:**
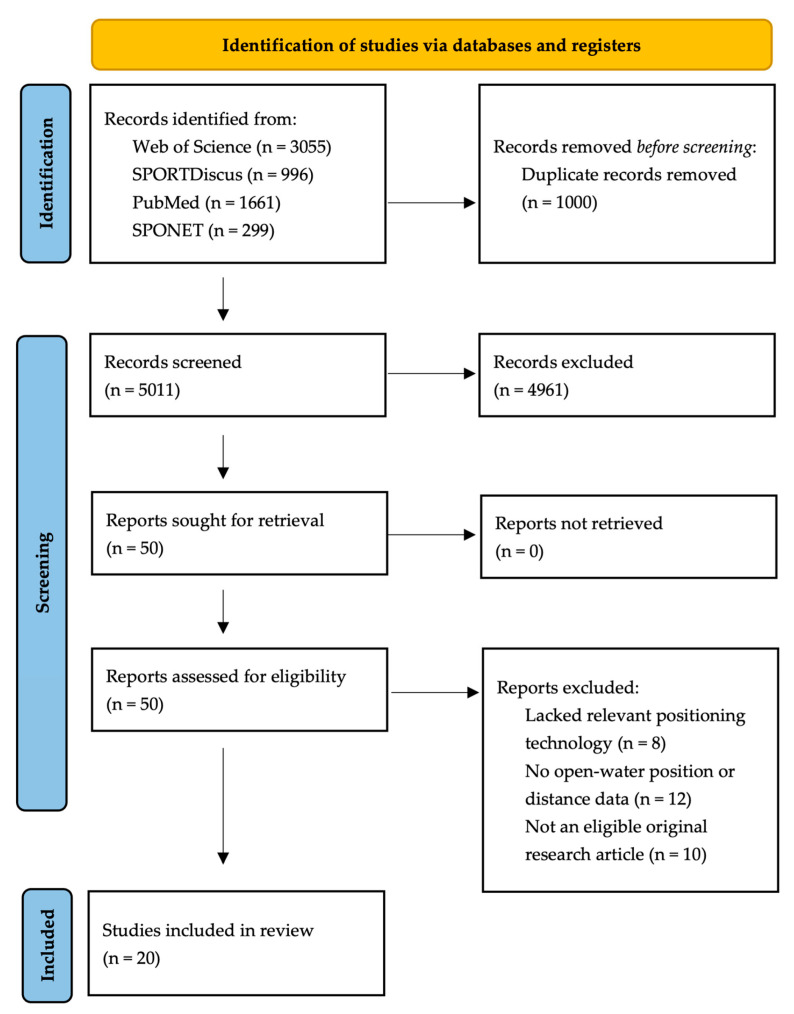
Visualisation of the systematic literature search process according to the PRISMA guidelines.

Two researchers (J.S. and M.W.) independently screened all records at the title and abstract level according to the predefined inclusion and exclusion criteria. Duplicates were initially identified and merged using reference management software (Zotero, version 7.0.32, Corporation for Digital Scholarship, Falls Church, VA, USA). During the screening phases, additional duplicates were manually reviewed and consolidated to ensure a non-redundant dataset. Records independently selected by both reviewers were directly forwarded for full-text retrieval. In cases of discordant selection, where a record was identified by only one of the two researchers, a third independent researcher (J.W.) evaluated the title and abstract of the record to determine its eligibility for further analysis. Full-text screening was conducted according to the predefined inclusion and exclusion criteria. The overall study selection process is shown in Figure 1.

Data extraction was performed by one researcher (J.S.) and reviewed by the co-authors (M.W. and J.W.). Emphasis was placed on methodological approaches to distance measurement in open-water sports and on insights derived from GNSS measurement parameters, with the aim of identifying implications for their application in open-water swimming within triathlon. Extracted variables included general study characteristics (sport, movement analysed, sample size, and performance level). Furthermore, detailed sensor specifications were recorded, including brand, sampling frequency (Hz), horizontal dilution of precision (HDOP), number of satellites, and sensor positioning. Analytical approaches based on sensor-derived data were systematically categorised into distance- and speed-related analyses, with further differentiation into derived metrics reflecting movement quality and tactical behaviour aspects. Movement quality metrics refer to GNSS-derived parameters that characterise the effectiveness, efficiency, or structure of movement patterns beyond absolute distance or speed measures, such as Velocity Made Good (VMG), activity distribution or speed zones. Tactical behaviour metrics comprise GNSS-derived measures that reflect discrete decision-related actions and race management behaviours within a competitive or task-specific context (e.g., manoeuvres, number of rides).

### 2.4. Assessment of Methodological Quality

The methodological quality of the included studies was assessed by one researcher (J.S.) using a custom-developed evaluation scheme, adapted from the GNSS reporting quality guidelines proposed by Malone et al. [7]. The evaluation encompassed six key reporting attributes: sampling frequency, HDOP, satellite information, device specifications, software and/or firmware details, and data processing procedures. Each attribute was rated on a three-point scale (0 = not reported, 1 = partially reported, 2 = fully reported and clearly described). “Partially reported” was assigned when a parameter was mentioned but lacked sufficient detail (e.g., satellite count reported only as a threshold value, general reference to data processing without specifying filtering procedures, or manufacturer reported without exact device model). The cumulative score (ranging from 0 to 12) was used to categorise studies into three quality tiers: low (<6 points), medium (6–8 points), and high (>8 points) reporting quality regarding GNSS-related methodological transparency. The scoring was based exclusively on the methodological information explicitly reported in the respective articles. The completed evaluation was independently reviewed by the co-authors (M.W., J.W.). No discrepancies requiring formal resolution were identified, as the criteria were based on reporting completeness.

### 2.5. Data Synthesis

As the review focused on methodological characteristics to develop a framework for continuous swim assessment in a triathlon, no statistical effect measures and further analyses were applied. GNSS-related parameters and analytical approaches were synthesised using a structured technical classification. Studies were grouped according to sampling frequency, satellite geometry reporting, sensor placement, validation status, and derived analytical constructs to enable systematic cross-disciplinary comparison of methodological architectures. All included studies were eligible for qualitative synthesis, and data were organised in standardised tables. Missing information was coded as “not reported”. Given the methodological heterogeneity and the absence of standardised outcome measures, no formal risk-of-bias tool and certainty-of-evidence evaluation were applied. Instead, GNSS-related reporting quality was assessed as an indicator of potential measurement- and processing-related bias relevant to the methodological aims of this review.

## 3. Results

### 3.1. Study Selection

A total of 6011 records were identified through database searching. After the removal of duplicates, 5011 studies remained for title and abstract screening. Of these, 4961 records were excluded based on predefined eligibility criteria. Consequently, 50 records were sought for retrieval, and 50 full-text articles were assessed for eligibility. Of these, 8 articles were excluded due to a lack of relevant technological approach of positioning technologies (only incidental use of positioning technology, Smartphone use, no mention of GNSS, Global Positioning System [GPS], or comparable tracking systems), 12 articles did not involve open-water environments with continuous movement trajectories, and 10 studies failed to meet the inclusion criterion of being original research articles published in eligible peer-reviewed journals. 20 studies were included in the qualitative synthesis.

Several studies identified during the search process demonstrated thematic relevance but did not fully meet the inclusion criteria due to insufficient detail regarding measurement technologies. These included works by Anastasiou et al. [18,19], Barlow et al. [20], Forsyth et al. [21], Gomes et al. [22], Pan & Sun [23], Perez-Turpin et al. [24], Silva et al. [25], and Wu et al. [15]. While these studies were excluded from the systematic analysis, they may nonetheless offer valuable contextual insights into the sport-specific application of GNSS technologies and associated performance metrics. Additionally, two surf-specific reviews by Mejuto et al. [13] and Farley et al. [12] provide in-depth analyses of the time–motion demands in surfing using objective GNSS-based measures.

### 3.2. Methodological Quality

The quality assessment revealed a broad spectrum of reporting standards among the included studies. Most studies were classified as either low (*n* = 8) or medium (*n* = 10) quality with respect to the reporting of GNSS-related methodological details. Only two studies met the criteria for high-quality reporting. Notably, critical parameters such as HDOP and the number of satellites used during data acquisition were rarely reported across the sample, indicating a general lack of transparency in essential GNSS metadata, see Table 2.

### 3.3. Study Characteristics

The 20 studies included in this review span a diverse range of open-water disciplines, reflecting the breadth of technological applications across aquatic sports. The majority focused on surfing (*n* = 7), followed by sailing (*n* = 4) and windsurfing (*n* = 2). Water skiing (*n* = 3) and kitesurfing (*n* = 2) were also represented, whereas only single studies addressed stand-up paddling (SUP) (*n* = 1) and swimming (*n* = 1). This distribution indicates a clear research emphasis on board- and sail-based sports, while highlighting the relative scarcity of sensor-based analyses in endurance disciplines such as open-water swimming and triathlon. Regarding the research approach, 14 studies analysed performance during actual races or competitions, while 6 studies applied experimental setups, often designed to evaluate training sessions or specific performance-relevant conditions under controlled scenarios. Across the included studies, methodological and technological heterogeneity was observed, particularly regarding GNSS device specifications, sampling frequencies, signal quality indicators (HDOP, satellite count), data-processing approaches, and the sport-specific movement characteristics of each aquatic discipline. This variability limits the comparability of outcomes across studies and precludes any meaningful meta-analysis, as no standardised effect metrics or compatible performance indicators were reported. Consequently, the present synthesis is restricted to a qualitative comparison of methodological approaches. The characteristics of the GNSSs employed in the included studies are summarised in Table 3. The table provides a comparative overview of device specifications, signal quality indicators, and their application across different open-water sports. For outcome comparison GNSS-based analytical approaches were extracted from the studies by sport. Table 4. provides a structured overview of the types of GNSS-derived distance, speed, movement quality, and tactical behaviour metrics reported across open-water sports. It illustrates the analytical focus adopted in different disciplines.

## 4. Discussion

### 4.1. Methods for Distance Measurement in Open-Water Sports

A diverse array of GNSS devices has been employed across open-water disciplines, ranging from research-grade multi-sensor units to commercially available wearables and embedded tracking platforms. Consistent with Malone et al. [7], each study ideally reports satellite geometry (number of satellites, HDOP), device specifications (brand, model, firmware versions), sampling frequency, and criteria for data inclusion and exclusion to ensure methodological transparency and facilitate cross-study comparisons. In the surveyed literature, Polar Systems (V800, G3) featured prominently in two investigations [31,38], while Catapult’s OptimEye S5 and MiniMax S4 appeared in surfing and swimming experiments [26,27]. GPSports units (HPISPU, SPI10) featured broadly across both experimental and race-based investigations [35,39,41], whereas in water skiing studies, LOCOSYS modules paired with Mighty GPS devices enabled dual measurement of both boat and skier [28,29,30]. TracTrac units were used in windsurfing races [11,34], and additional deployments included SurfTraX [36,37], VX Sport VX110 [42], Mylaps X5 [40], and Qstarz BT-Q100P [43]. Several studies provided only analytical results without specifying hardware [14,32,33].

Sampling frequencies varied considerably across the literature: approximately 1 Hz was used in several studies [28,29,30,31,35,43], while mid-range rates of >1–5 Hz appeared in others [11,28,29,30,32,34,38,42]. High-frequency sampling (>5–15 Hz) characterised investigations demanding fine-grained tactical insight [26,27,36,37,39,41]. Notably, several studies did not report their sampling rates at all [14,33,40]. It should be noted that commercially available GNSS loggers with substantially higher update rates (e.g., 25 Hz) are available. However, such systems have not yet been applied in the context of open-water sport performance analysis. From a measurement perspective, sampling frequency determines the temporal resolution of derived speed and distance metrics. Higher update rates may improve detection of short-duration pacing fluctuations and rapid directional changes. However, they may also increase susceptibility to signal noise if satellite geometry and data filtering are not adequately controlled. Conversely, lower sampling rates may smooth transient variations and underestimate short-term speed changes, potentially affecting the interpretation of tactical dynamics.

Sensor placement reflected sport-specific and technical constraints: upper-back or scapular mounting under wetsuits dominated surfing research [11,26,35,36,39,41,43]; wrist or arm mounts (e.g., Polar V800) appeared in kitesurfing, windsurfing and surfing [31,37,38]; head-mounted units integrated into swim caps or helmets were trialled in swimming and water skiing [27,28,29,30]. Board- and boat-embedded units provided vehicle-level trajectory data in water skiing [28,29,30], windsurfing [14], and sailing [33,40]. Several studies omitted details on sensor placement or did not specify the exact region on the body [11,32,34,42]. Sensor placement may substantially influence signal stability and derived speed estimates, particularly in aquatic environments where partial submersion, body rotation, and changes in antenna orientation occur. Head-mounted systems, for example, may reduce signal loss during swimming. The optimal sensor position therefore depends on the specific movement characteristics and technical demands of the respective sport discipline.

Accurate and precise positional fixes depend on both satellite count and signal geometry. Ideal horizontal dilution of precision (HDOP < 1.0) indicates optimal satellite spread [7]. Only three studies reported HDOP values: 0.96 ± 0.29 for Catapult S5 in female surfers [26], 1.10 ± 0.18 for Catapult MiniMax S4 in swimmers [27], and 0.95 ± 3.70 for SurfTraX in male surfers [37]. Satellite counts, essential for three-dimensional fixes (minimum of four) [44], were documented in four investigations: 13 ± 1 satellites [26], 9 ± 1 [27], at least 8 [38], and more than 3 [41]. In open-water settings, satellite availability and HDOP values may be influenced by environmental constraints such as wave motion, water surface multipath reflections, and surrounding shoreline structures. These factors can affect positional accuracy and precision depending on the specific measurement configuration and movement context.

Validation data remain scarce, with only a few studies reporting formal reliability and accuracy and precision assessments. For instance, Beanland et al. [27] established criterion validity by showing GPS-derived velocities did not differ significantly from video-based measurements in freestyle, breaststroke, and butterfly, with SEM values between 0.12 and 0.18 m·s^−1^. SurfTraX reliability was confirmed via inter-unit ICCs for speed (0.30–1.00), distance (0.23–0.92), and time (0.18–0.98) [36]. Mylaps X5 sensors averaged 2.5 m location error [40]. Secomb et al. [42] reported CVs of 4.6% for distance and 2.9% for maximal speed estimation for the VX Sport VX110 Log. However, validation outcomes are difficult to compare directly, as studies differed in reference standards (e.g., video-based criterion measures vs. device-based reliability assessments), environmental exposure, and reporting of data processing procedures. Information on smoothing algorithms, filtering thresholds, or signal-cleaning procedures was rarely provided, limiting the metrological interpretability of high-frequency speed and distance estimates.

Table 5 summarises the key GNSS-related methodological factors identified across the reviewed studies and their potential implications for open-water sports analysis.

Collectively, these findings underscore the heterogeneous nature of GNSS-based distance measurement in open-water sports. To advance both precision and comparability, future research must adhere to standardised reporting of device characteristics, sampling protocols, sensor placements, and satellite geometry metrics. Beyond transparent reporting, systematic investigation is needed to determine how these technical parameters relate to measurement accuracy and limits of agreement under ecologically valid open-water conditions.

### 4.2. Derivable Insights from GNSS Measurement Parameters in Open-Water Sports

GNSS-based systems determine an athlete’s position via triangulation, measuring the ranges to multiple satellites and computing latitude/longitude fixes. The total distance covered is then obtained by summing the Euclidean displacements between successive fixes after transforming geographic latitude–longitude coordinates into an appropriate planar coordinate system (e.g., via great-circle approximations such as the Haversine formula), with speed derived as the time-derivative of that distance. Alternatively, Doppler-shift measurements of the satellite carrier signal yield near-instantaneous velocity estimates, which can be integrated over time to calculate distance [7]. Distance covered, either in total or over key race segments, and the actual course taken are the most relevant parameters in GNSS-based open-water studies. Across the examined sports, GNSS-derived distance metrics capture discipline-specific movement patterns. For example, Caraballo et al. [11] analysed 159 Olympic Laser-class sailors (92 men, 67 women) and showed that elite competitors travel a shorter distance on the upwind leg compared to broad-reach and downwind sections. In surfing, Barlow et al. [26] reported that higher-ability surfers allocate a greater proportion of their total distance to actual wave riding, while spending comparatively less on non-productive activities such as unsuccessful paddling attempts, wipeouts, or suboptimal wave selection. This pattern suggests more efficient wave selection strategies and a higher number of effective rides among elite performers. In RS:X windsurfing, Caraballo et al. [14] reported that top-tier athletes covered shorter distances on upwind courses than their lower-ranked peers, reflecting more direct tactical lines. Although open-water distance metrics are inherently influenced by environmental conditions (e.g., wave height, wind strength, and direction), covered distance consistently differentiates between high- and lower-level performers across disciplines. Similarly, recent GNSS-based analyses in elite 10 km open-water swimming have demonstrated measurable differences in actual race distance and trajectory selection between athletes, indicating that subtle variations in line choice and positioning contribute to cumulative distance disparities during competition. Notably, the eventual race winner covered the shortest effective race distance, suggesting that more economical navigation and reduced lateral deviation may represent performance-relevant determinants in mass-start open-water events [6].

Speed is the primary kinematic parameter derived from GNSS data, but researchers have also examined more nuanced speed-related metrics to capture sport-specific demands. Across open-water sports, speed metrics were reported at multiple aggregation levels, including mean and maximal speeds, segment-specific speeds, and activity- or phase-specific measures, allowing comparisons across performance levels, race formats, and environmental conditions. Caraballo et al. [14] examined the influence of performance level and sex on velocity, distance covered, and manoeuvre-related variables during the 2019 RS:X Class World Cup in windsurfing. Their findings indicated that higher-level athletes achieved significantly greater mean speeds across all course segments including upwind, reaching, and downwind, highlighting the impact of skill level on race performance metrics. In swim-specific applications, GNSS data have predominantly been analysed using predefined splits or segment-based approaches rather than fully continuous pacing models. For example, Wu et al. [15] monitored eight trained male triathletes over four equal swim splits and reported no significant differences in mean swim speed between splits or overall distance segments, indicating consistent pacing across the entire swim leg. In contrast to these findings, earlier triathlon studies also using fixed splits have produced inconsistent pacing profiles. As already shown Vleck et al. [2,3], reported positive pacing patterns in an Olympic-distance race. However, no consistent tracking was used. It should be noted that Wu et al. [15] analysed top age-group athletes across three distances (sprint, Olympic, and middle-distance triathlon) whereas Vleck et al. [2,3] analysed elite triathletes competing over the Olympic distance. These findings illustrate the constraints of segment-based analyses for capturing detailed intra-race pacing variations. Beanland et al. [27] demonstrated that sub-elite swimmers’ velocities derived from GPS closely matched those obtained via video split analysis for both freestyle and breaststroke. No significant differences were observed across the 21 athletes, supporting GNSS as a reliable tool for high-resolution swim monitoring. Beyond validation contexts, recent open-water competition data demonstrate that continuous GNSS-derived speed profiles can resolve intra-race intensity fluctuations and lap-specific changes in race dynamics during elite 10 km events [6]. Such analyses reveal performance-relevant variations in speed distribution that remain undetectable in coarse split-based assessments. However, both cumulative distance and short-term speed estimates are sensitive to sampling frequency, signal quality, and environmental interference, which may differentially affect the resolution of pacing dynamics and the detection of rapid tactical actions. Evidence from land-based team sports suggests that sampling frequency, satellite availability, and environmental obstructions can influence both distance and speed estimates, particularly during high-speed or multidirectional movements [45].

Conceptually, GNSS-derived distance and speed constitute the primary kinematic descriptors across open-water sports. These variables are rarely interpreted in isolation. Instead, they form the basis for derived analytical domains. Transformations of distance and speed are used to characterise movement quality, whereas event-based segmentations of these signals capture tactical behaviour. Movement quality metrics describe how effectively athletes convert distance and speed into goal-oriented progression. In sail-powered sports, VMG integrates both speed and direction relative to the wind or a navigation mark, quantifying net progress along the optimal course [11,14,32,33,43]. VMG accounts for trade-offs between sailing angle and velocity [43]. Caraballo et al. [11] found that the most successful Olympic sailors, both men and women, achieved higher VMG values on upwind and downwind legs than their lower-ranked counterparts, underscoring VMG’s utility in assessing navigation efficiency. Especially in surfing, movement quality was further characterised using activity distribution and speed zone metrics. Activity distribution metrics quantify the allocation of time across distinct activity states (e.g., sitting, paddling, and wave riding in surfing) [26,31,38,39]. While Barlow et al. [26] linked activity distribution to competitive performance in surfing, Fernandez-Gamboa et al. [38] and O’Neill et al. [39] primarily applied these metrics to characterise external load and physiological demand. Specifically, Barlow et al. [26] reported that greater competitive success was associated with a higher proportion of time and distance spent wave riding, whereas increased sitting time was related to poorer heat placements. Speed zone analyses were used across multiple investigations [35,39,41] to distinguish movements (paddling and wave-riding) and to link external intensity profiles with internal markers such as %HR_max_. In water skiing and sailing, movement quality was assessed using outcome- or path-based measures, including successful turns or buoys completed [28,30] and course coverage (distance sailed relative to course distance) [40]. In kitesurfing, movement direction was characterised by the hauled wind angle, describing directional efficiency during upwind sailing [31]. GNSS-based analyses in elite 10 km open-water swimming further extended movement quality assessment by integrating continuous speed data with individual critical velocity, enabling quantification of intensity distribution and cumulative race distance in relation to performance outcomes [6].

Tactical behaviour reflects how athletes’ structure and sequence actions during competition and is derived from discrete segmentations of distance and speed data. In sail-powered sports, the number of manoeuvres complements distance and speed metrics by capturing tactical decision-making on course [11,14,32,33,40,43]. Manoeuvres, namely tacks (turning the bow through the wind on upwind legs) and jibes (turning the stern through the wind on downwind or broad-reach legs) involve shifting the sails from one side of the boat to the other and correspond to course angles of approximately 45° (upwind), 120° (broad reach) and 180° (downwind) relative to the wind [11]. Analyses have yielded mixed findings. Caraballo et al. [11] found no significant differences in total manoeuvre counts across upwind, broad-reach or downwind legs when comparing the full Laser-class sample or sex-specific groups, indicating that manoeuvre frequency alone did not distinguish performance level. In contrast, in RS:X windsurfing, Caraballo et al. [14] detected that top-tier sailors performed significantly fewer manoeuvres on the upwind leg than lower-ranked competitors, suggesting that reduced manoeuvring, thereby minimising speed losses, contributes to higher performance. Manoeuvre analysis thus elucidates key tactical decision points by quantifying their frequency, timing and associated speed fluctuations. In surfing, the number of rides was used as a tactical event metric and was positively associated with heat placement, with a higher number of rides linked to better competitive outcomes [26]. In kitesurfing, the number of beats quantified tactical event structure, defined as straight sailing segments between turns during course racing [31].

**Figure 2:**
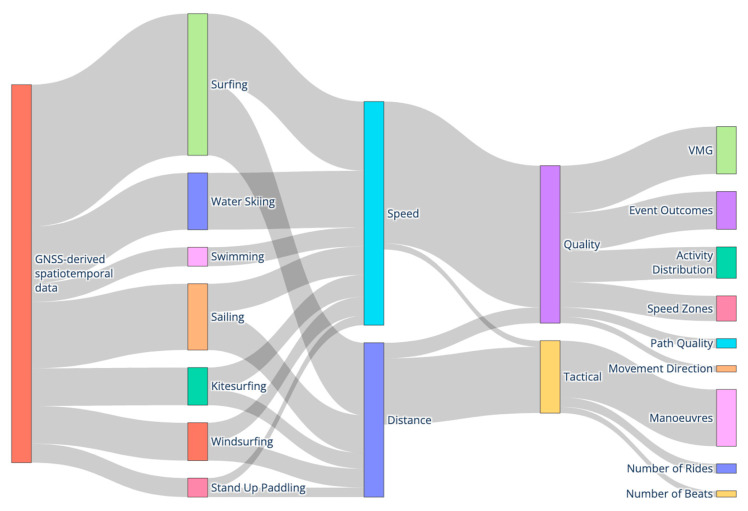
Conceptual Sankey diagram illustrating how GNSS-derived distance and speed metrics are transformed into movement quality and tactical performance indicators across open-water sports. Grey bands represent the number of studies linking categories, with wider bands indicating a greater number of studies.

Figure 2 provides a conceptual synthesis of the GNSS-derived analytical approaches summarised in Table 4. The Sankey diagram is structured in three analytical layers. The left column represents the open-water disciplines included in this review. The central nodes (“Distance” and “Speed”) depict the primary GNSS-derived kinematic variables. These feed into derived analytical domains shown in the next layer (“Quality” and “Tactical”), which reflect how raw positional data are transformed into performance-relevant constructs. The terminal nodes on the right illustrate specific metrics reported in the literature (e.g., VMG, speed zones, manoeuvres, number of rides). Link widths indicate the presence of analytical approaches across studies. The figure visualises methodological pathways from raw GNSS data to applied performance metrics across disciplines.

Accordingly, the integration of continuous distance and speed profiling represents a necessary step toward more precise modelling of pacing behaviour and tactical positioning in mass-start open-water events. Despite the methodological heterogeneity across disciplines, certain GNSS-derived variables may be less sensitive to moderate differences in sampling frequency and device configuration. Total distance covered and large-scale trajectory reconstruction are theoretically less dependent on high-frequency sampling and short-term signal stability. In contrast, instantaneous speed fluctuations, acceleration-based metrics, and fine-grained intensity classifications may be more susceptible to sampling rate, filtering procedures, and environmental exposure, as these variables rely on derivatives of positional data [7]. These potential differential sensitivities should be considered when interpreting and transferring GNSS-based performance indicators across disciplines.

### 4.3. Developing a Data-Driven Swim Leg Strategy for Triathlon

Swimming in a triathlon is subject to highly variable course conditions, especially currents and weather [46], and encompasses distances from approximately 250 m (team relay, super-sprint) to 1500 m (Olympic distance), with in-race factors such as drafting and wetsuit use further modulating performance [1,47]. Vleck et al. [3] demonstrated significant speed differentials between the start and first buoy (222 m) compared to subsequent segments in male and to the next segment from the first buoy (222 m) to the next buoy (496 m) in female Olympic-distance triathletes, underscoring the importance of front-pack swim positioning for successful transition into the lead cycling group [1,48]. Physiologically, the intermittent nature of triathlon imposes unique demands across swim, bike and run legs [49], yet existing analyses rely on sparse buoy-based splits calibrated with ropes and video timing, thereby neglecting the actual distance traversed and fine-grained pacing dynamics [3,15]. In contrast, recent GNSS-based analyses of elite 10 km open-water competition demonstrated that continuous tracking can quantify both actual race distance and intra-race speed distribution, revealing that the race winner covered the shortest effective distance and strategically delayed sustained supra-threshold efforts until the final lap [6]. In that study, continuous 1 Hz head-mounted GNSS tracking combined with LOESS-based smoothing enabled detailed spatiotemporal pacing analysis under official race conditions. Although signal artefacts and data loss were reported in a subset of recordings, the approach demonstrated the practical feasibility of combining positional tracking with physiological benchmarking in elite open-water racing. Insights from GNSS research in open-water sports reveal that board- and wave-based disciplines (surfing, SUP) routinely employ high sampling rates of 10–15 Hz to capture rapid, multidirectional movements [26,36,41], whereas large-scale movement sports such as sailing and kitesurfing operate at lower rates of 1–5 Hz [11,34]. Sensor placement also follows discipline-specific patterns: surfing and SUP units sit beneath the wetsuit at the upper back/scapula [26,36,41], whereas swimming in experimental pool settings have been mounted on the head to minimise underwater signal loss [27]. These findings suggest that both sampling frequency and mounting location critically affect data fidelity in aquatic environments. In addition to distance and speed, GNSSs enable the extraction of performance-related variables such as manoeuvres, VMG, and speed zones in the analysed open-water sports. Although not all these metrics can be directly transferred to triathlon, they serve as a conceptual reference point for advancing sport-specific analyses of pacing, efficiency, and transitions between disciplines.

The reviewed literature indicates that GNSS-based systems have become a standard tool for quantifying open-water and race-specific metrics. However, ensuring the measurement accuracy and precision of these data requires integration with additional sensor modalities and rigorous calibration protocols. Several methodological challenges identified in the reviewed literature require explicit consideration when translating these approaches to triathlon swim analysis. Repeated submersion during stroke cycles may lead to transient signal loss, while multipath reflections at the water surface can introduce positional noise even under favourable satellite geometry. Moreover, limited reporting of filtering and smoothing procedures complicates the interpretation of high-frequency speed estimates. Addressing these constraints is essential for developing a robust and practically applicable GNSS-based framework.

In response to these methodological constraints, we propose the following framework for GNSS-based swim leg analysis in triathlon: (1) employ a head-mounted GNSS sensor to reduce submersion-induced signal dropouts [27]; (2) sample at >5 Hz to resolve microvariations in pacing and drafting interactions; (3) ensure positional accuracy and precision via HDOP < 1.0 [7] and a minimum of four satellites in view [44]; and (4) compute true distance covered alongside striking points (buoy and round comparison depending on the length of the race).

While head-mounted placement reduces prolonged signal loss due to full submersion, transient attenuation during stroke cycles and head rotation may still occur. Such short-term dropouts should be anticipated and corrected through transparently reported post-processing procedures (e.g., interpolation or smoothing). Where feasible, integration with complementary sensors such as IMU or video-based tracking can further enhance measurement robustness under dynamic open-water conditions. Even under optimal HDOP and satellite availability, surface-related multipath effects remain a challenge in open-water settings and require careful signal evaluation.

For real-world implementation, practical considerations such as waterproof housing, secure fixation within swim caps, hydrodynamic drag, athlete comfort, and compliance with competition regulations must also be addressed. Evidence from elite open-water competition further indicates that such high-resolution GNSS-derived distance and speed profiling can detect performance-relevant differences in navigation efficiency and intensity distribution that are directly associated with race outcomes [6]. This approach will enable coaches and researchers to derive targeted, data-driven swim strategies that account for both external conditions and in-race tactics.

### 4.4. Limitations

Although the search was conducted across four major databases, relevant studies indexed in other repositories or published in non-English languages may have been missed, potentially limiting the comprehensiveness of the review. The decision to exclude studies relying solely on inertial sensors (e.g., IMU or accelerometers) as well as studies with thematic relevance but insufficient detail on measurement technologies may have omitted potentially valuable insights, particularly in disciplines where GNSS signals are intermittent or unavailable. Nevertheless, the final sample reflects the current evidence base available under the predefined eligibility criteria. The methodological quality and reporting detail of GNSS-related parameters varied substantially across included studies. Inconsistent or missing data regarding key variables such as HDOP, satellite count, or sampling frequency may limit the interpretability and comparability of results. The review may be affected by publication bias, as studies reporting successful or technically robust GNSS applications are more likely to be published than studies with inconclusive or unfavourable measurement results. Selection bias cannot be fully excluded despite predefined eligibility criteria. A formal risk-of-bias assessment was not feasible due to substantial methodological heterogeneity. Conventional tools to detect publication bias were not applicable without meta-analytic pooling.

Future research should aim to establish and adopt standardised reporting guidelines for GNSS-based studies in sport science, including parameters such as HDOP, satellite count, sampling frequency, firmware/software specifications, and data processing steps to improve methodological transparency and data comparability across studies. Combining GNSS with additional sensor technologies such as IMU could enable a more comprehensive assessment of external and internal load, particularly in complex movement patterns typical of open-water sports. Swimming and the swim leg in a triathlon remain underexplored in the context of GNSS technology. Future studies should address the feasibility, accuracy and precision, and practical applications of GNSS-based monitoring in open-water swim environments, including aspects such as race analysis, pacing strategies, and the influence of environmental factors like currents and water temperature on positional data quality and athlete performance.

## 5. Conclusions

This systematic review highlights substantial methodological heterogeneity and inconsistent reporting standards in GNSS-based analyses of open-water sports. While GNSS-based technologies are widely applied across disciplines such as sailing and surfing, their use in swimming and triathlon remains limited. Across the reviewed literature, GNSS data were analysed using a wide range of sport-specific approaches, extending beyond basic distance and speed metrics to derived indicators of movement quality and tactical behaviour. Building on these insights, the proposed framework outlines essential technical considerations for reliable GNSS-based swim leg assessment, emphasising high sampling rates, the control of accurate and precise positioning, and robust data handling applied to triathlon swimming. Integrating GNSS with complementary sensor systems may enhance data accuracy and precision and expand analytical possibilities.

## Figures and Tables

**Table 1:** Inclusion/Exclusion criteria based on the PICO(S) scheme.

Item	Inclusion	Exclusion
Population	Open-water sports such as triathlon, open-water swimming, sailing, windsurfing, kitesurfing, surfing, stand-up paddling	Pool swimmers, Paralympic athletes, animal studies, or non-sporting subjects and other non-open-water sports
Intervention	Application or validation of GNSS sensor-based technologies to analyse continuous movement under sport-specific open-water conditions	Studies using only inertial sensors (no positioning), indoor protocols, non-continuous movements
Comparison	Different GNSSs and measurement accuracy and precision	Studies without any comparative or analytical component
Outcome	Data quality (accuracy and precision, validity, reliability), practical feasibility, technological suitability for open-water sports analysis	Studies lacking performance-relevant outcomes or missing evaluation of technological aspects
Study Type	Peer-reviewed, full-text, original research articles published in English in Sport Sciences or Engineering	Reviews, conference abstracts, non-peer-reviewed works, or other publications outside the defined fields

**Table 2:** Quality criteria for the GNSS usage in the articles including the mention of Sampling size, HDOP, Number of satellites, Device information, Software/Firmware information, and Data processing information.

Study	Sampling	HDOP	Satellite	Device	Software/ Firmware	Data Processing	Score	Quality
Barlow et al. [26]	2	2	2	2	2	2	12	high
Beanland et al. [27]	2	2	2	2	2	2	12	high
Bray-Miners et al. [28]	2	0	0	2	2	0	6	medium
Bray-Miners et al. [29]	2	0	0	2	2	2	8	medium
Bray-Miners et al. [30]	2	0	0	2	2	2	8	medium
Caimmi & Semprini [31]	2	0	0	2	2	0	6	medium
Caraballo et al. [11]	2	0	0	1	2	0	5	low
Caraballo et al. [32]	2	0	0	0	2	0	4	low
Caraballo et al. [14]	0	0	0	0	2	0	2	low
Caraballo et al. [33]	0	0	0	0	2	0	2	low
Chun et al. [34]	2	0	0	1	2	0	5	low
Farley et al. [35]	2	0	0	2	2	1	7	medium
Farley et al. [36]	2	0	0	2	0	0	4	low
Farley et al. [37]	2	2	0	2	2	0	8	medium
Fernandez-Gamboa et al. [38]	2	0	1	2	0	0	5	low
O’Neill et al. [39]	2	0	0	2	2	0	6	medium
Philippe at al. [40]	0	0	0	2	0	0	2	low
Schram et al. [41]	2	0	1	2	2	1	8	medium
Secomb et al. [42]	2	0	0	2	2	0	6	medium
Winchcombe et al. [43]	2	0	0	2	2	0	6	medium

Note: Studies were rated on a three-point scale (0 = not reported, 1 = partially reported, 2 = fully reported and clearly described). The cumulative score (0–12) was used to classify studies into three quality tiers regarding GNSS-related methodological transparency: low (<6 points), medium (6–8 points), and high (>8 points).

**Table 3:** List of the included articles with Reference, Sport, Movement, Sample size, Performance level, Sensor (including the brand), the Sampling frequency (Hz), HDOP, Number of satellites and Sensor placing.

Reference	Sport	Movement	Sample	Performance Level	Sensor (Brand)	Sampling Frequency [Hz]	HDOP	Number of Satellites [Mean ± SD] (Range)	Sensor Placing
Caimmi & Semprini [31]	Kitesurfing	Race	5 (5 m)	High-level (ranked in the best positions in Championship)	G3 GPS monitor (Polar Electro, Oy, Kempele, Oulu, Finland)	1	–	–	Proximal third of the right arm
Caraballo et al. [32]	Kitesurfing	Race	42 (35 m, 7 w)	Olympic	–	5	–	–	Placed on the sailor
Caraballo et al. [11]	Sailing	Race	159 (92 m, 67 w)	Olympic (participated in a World Cup)	– (TracTrac, Lyngby, Denmark)	5	–	–	Placed on the sailor
Caraballo et al. [33]	Sailing	Race	203 (121 m, 82 w)	Elite (competed in an international regatta)	–	–	–	–	Placed on the sailor’s boat
Philippe et al. [40]	Sailing	Race	21 (21 m)	Professional (international; collective experience included 59 Tour de France campaigns)	Mylaps X5 GPS (Mylaps, Haarlem, Netherlands)	–	–	–	Boat placed
Winchcombe et al. [43]	Sailing	Race	11 (11 m)	Elite (World Sailing ranking ranged from top 3 (*n* = 2), 4 to 50 (*n* = 3), 51 to 100 (*n* = 2), or >100 (*n* = 4)	BT-Q1000P (Qstarz International Ltd., Taipei, Taiwan)	1	–	–	Worn in their life jacket pocket
Schram et al. [41]	SUP	Race	10 (6 m, 4 w)	Elite (national top 10 or international top 24)	GPSports HPISPU (GPSports Systems Ltd., Canberra, Australia)	15	–	>3	In a waterproof zip-lock bag on the front or back pocket of the hydration packs worn on the chest
Barlow et al. [26]	Surfing	Race	22 (22 w)	Train rigorously and compete regularly at high level surfing competitions	Catapult S5 (Catapult Sports, Melbourne, Australia)	10	0.96 ± 0.29	13 ± 1 (11–15)	Inside two knotted nitrile gloves inside the wetsuit between the shoulder blades in-line with the spine
Farley et al. [35]	Surfing	Race	12 (12 m)	National level (current top 30 ranked surfers in New Zealand)	SPI10 Sports Performance Indicator (GPSports Systems Ltd., Canberra, Australia)	1	–	–	Under the wetsuit of the subject around the upper thoracic vertebra and scapula into a watertight sealed bag
Farley et al. [36]	Surfing	Race	10 (–)	Competitive	SurfTraX (Southport, Australia)	10	–	–	On the upper vertebrae, between the scapulars and held in position in the back pouch of the chest zip wetsuit
Farley et al. [37]	Surfing	Race	41 (41 m)	Competitive (national ranked)	SurfTraX (Southport, Australia)	10	0.95 ± 3.7	–	In a sealed arm strap tightened around the biceps with the unit positioned on the triceps, or, if the surfer wore a chest zip wetsuit, on the upper vertebrae held in position in the back pouch of the suit
Fernandez-Gamboa et al. [38]	Surfing	Race	10 (–)	Competitive (minimum of seven years of experience)	Polar Electro V800 (Polar Inc., Kempele, Oulu, Finland)	2.4	–	>8	Wrist
O’Neill et al. [39]	Surfing	Experiment	10 (10 m)	Recreational (five years surfing experience)	GPSports HPISPU (GPSports Systems Ltd., Canberra, Australia)	15	–	–	In a waterproof zip tight bag worn in the supplied vest
Secomb et al. [42]	Surfing	Experiment	15 (15 m)	Regional level (have a minimum competitive experience in at least 3 competitions at this level)	VX Sport VX110 Log (Visuallex Sport International Ltd., Lower Hutt, New Zealand)	4	–	–	Worn
Beanland et al. [27]	Swimming	Experiment	21 (12 m, 9 w)	Sub-elite (regular involvement in high level swimming—five sessions per week—and achieved an Australian State level qualifying time in the past swimming season)	Catapult minimax S4 (Catapult Sports, Melbourne, Australia)	10	1.1 ± 0.18 (freestyle); 1.25 ± 0.3 (breaststroke); 1.53 ± 0.36 (butterfly)	9 ± 1	On the head (vertical axis of the accelerometer directly inferior to the inion, in line with the sagittal suture)
Bray-Miners et al. [28]	Water skiing	Experiment	6 (6 m)	Advanced (be able to ski two passes of a slalom course on four different slalom skis in one test day)	Subject: LS20032 (LOCOSYS Technology Inc., Xizhi City, Taiwan) Boat: BG-331RGTGT (Mighty GPS, Toronto, ON, Canada)	Subject: 5; Boat: 1	–	–	Subject: helmet-mounted (wrapped in plastic and placed between the helmet shell and foam lining) Boat: in the boat (did not require any modifications)
Bray-Miners et al. [29]	Water skiing	Experiment	6 (6 m)	Advanced (be able to ski two passes of a slalom course on four different slalom skis in one test day)	Subject: LS20032 (LOCOSYS Technology Inc., Xizhi City, Taiwan) Boat: BG-331RGTGT (Mighty GPS, Toronto, ON, Canada)	Subject: 5; Boat: 1	–	–	Subject: helmet-mounted (wrapped in plastic and placed between the helmet shell and foam lining) Boat: in the boat (did not require any modifications)
Bray-Miners et al. [30]	Water skiing	Experiment	6 (6 m)	Advanced (be able to ski two passes of a slalom course on four different slalom skis in one test day	Subject: LS20032 (LOCOSYS Technology Inc., Xizhi City, Taiwan) Boat: BG-331RGTGT (Mighty GPS, Toronto, ON, Canada)	Subject: 5; Boat: 1	–	–	Subject: helmet-mounted (wrapped in plastic and placed between the helmet shell and foam lining) Boat: in the boat (did not require any modifications)
Caraballo et al. [14]	Windsurfing	Race	53 (28 m, 25 w)	Olympic (final of the RS:X class World Cup held in 2019 in Marseille, France)	–	–	–	–	Placed on the board
Chun et al. [34]	Windsurfing	Race	44 (44 m)	High and low performance (data from 2020 World Cup series in Enoshima, Japan)	– (TracTrac, Lyngby, Denmark)	5	–	–	–

Note: – indicates information not reported.

**Table 4:** Summary of GNSS-based analytical approaches for distance, speed, movement quality, and tactical behaviour in open-water sports.

Sport	Distance	Speed	Quality	Tactical
Sailing	Section (upwind, broad reach, running) and race format (NS, CR) [11,33,40]	Overall and segment-specific in group-based comparisons across race formats (NS, CR) [40]	Integrated speed-based metrics (VMG) by course section (upwind, broad reach, running), performance group, sex, wind speed, and race format; path-based metrics as course coverage (distance sailed relative to course distance) for race formats [NS, CR] and groups [11,33,40,43]	Manoeuvre counts (total, per heat), by course section (upwind, broad reach, running), race format (NS, CR), manoeuvre type (tacks, gybes), sex, performance level, wind speed, and race context [11,33,40,43]
Surfing	Activity (paddling, wave riding, sitting), wave-specific, and per heat or time interval [26,35,36,37,38,39,42]	Aggregation levels (wave-specific, activity-specific [wave riding], heat-based, time-segmented), and speed zones [26,35,36,37,38,39,42]	Activity-based metrics (time spent paddling, wave riding, sitting, stationary, active), and time across speed zones [26,35,38,39]	Counts of activity-related events (number of rides) [26]
Windsurfing	Start-line distance and by section (upwind, reaching, downwind) under varying wind conditions [14,34]	Race phases (pre-start, start, segment-specific speed [upwind, reaching, downwind]) under varying wind and performance conditions [14,34]	Integrated speed-based metrics (VMG) across race legs (upwind, reaching, downwind), by performance level and sex [14]	Counts of manoeuvres by course section (upwind, reaching, downwind), performance level and sex [14]
Kitesurfing	Per regatta and section (upwind, downwind, beam reach) [31,32]	Overall and segment-specific for directions (hauled, reaching, broad reach), and round- and performance-level [31,32]	Activity-based and directional metrics (duration of “beats”, hauled wind angle) and integrated speed-based metrics (VMG) across course sections and performance levels [31,32]	Counts of “beats” and manoeuvres by course section (upwind, downwind, beam reach) and performance level [31,32]
Water skiing	–	Actions (turns or cutting phases), including individual-level peak speed profiles [28,29,30]	Outcomes limited to event-based success metrics [28,30]	–
SUP	Per time unit (per minute) and per sex [41]	Overall per sex [41]	Time across speed zones [41]	–
Swimming	–	Validation for stroke conditions (freestyle, breaststroke, butterfly) [27]	–	–

Note: – indicates information not reported. VMG = Velocity Made Good; NS = nautical stadium; CR = coastal raid; SUP = stand-up paddling.

**Table 5:** Key GNSS-related methodological factors and their implications for open-water sports analysis.

Methodological Factor	Reported Values	Implication
Sampling frequency	Frequencies ranged from low (1 Hz) to high (>5–15 Hz), with several studies not reporting sampling frequency	Lower frequencies may smooth transient variations, whereas higher frequencies may increase temporal resolution but may also increase susceptibility to signal noise
Sensor placement	Placement varied across disciplines, including upper-back, wrist- or arm-mounted, head-mounted, and board- or boat-embedded configurations	May affect signal continuity and derived speed estimates; aquatic motion and partial submersion may reduce signal stability
Satellite geometry	HDOP and satellite counts were inconsistently reported; HDOP values included 0.95 ± 3.70, 0.96 ± 0.29, and 1.10 ± 0.18, while satellite counts ranged from >3 to 13 ± 1	May be influenced by environmental constraints (e.g., wave motion, water-surface multipath reflections, shoreline structures); may affect positional accuracy and precision depending on the measurement configuration and movement context
Validation	Validation evidence was limited and heterogeneous, including SEM values of 0.12–0.18 m·s^−1^ for swimming velocity, inter-unit ICCs of 0.18–1.00 across surfing speed, distance and time, a mean positional error of 2.5 m in sailing, and CVs of 4.6% for distance and 2.9% for maximal speed in surfing	Limits direct comparability and metrological interpretability

## Data Availability

The original contributions presented in this study are included in the article. Further inquiries can be directed to the corresponding author.

## Abbreviations

The following abbreviations are used in this manuscript:

GNSS	Global Navigation Satellite System
GPS	Global Positioning System
HDOP	Horizontal Dilution of Precision
IMU	Inertial Measurement Units
SUP	Stand-up paddling
VMG	Velocity Made Good
